# Religiosity and Perceived Social Support Enhance Older Adults’ Resilience after the 2016 Flood

**DOI:** 10.1093/geroni/igab046.3339

**Published:** 2021-12-17

**Authors:** Katie Cherry, Matthew Calamia, Emily Elliott, Angelina Cantelli

**Affiliations:** 1 Louisiana State University, Louisiana State University/Baton Rouge, Louisiana, United States; 2 Louisiana State University, Baton Rouge, Louisiana, United States

## Abstract

In 2016, catastrophic flooding destroyed homes and property across south Louisiana. This study is part of a larger program of research that addresses the role of prior hurricane and flood experiences on current health and well-being in later life. Participants were predominately middle-aged and older adults who varied in current and prior severe weather experiences (M age=49.6 years, age range 18-88 years). All were tested during the immediate aftermath of the 2016 flood (Wave 1; N=223) and most participated in a follow-up assessment 9 (+/- 3) months later (Wave 2; N=202). Cherry et al. (2021) reported that greater flood stressors at Wave 1, such as displacement, flood-related losses, and damage to homes and property, were associated with more symptoms of post-traumatic stress disorder (PTSD). In this study, we tested the hypothesis that age, religiosity, and perceived social support would be positively associated with post-flood resilience at the Wave 2 follow-up. Results indicated that age was positively associated with religiosity and resilience, and negatively correlated with symptoms of PTSD. Additionally, faith community involvement, non-organizational religiosity, and religious beliefs and practices were all significantly correlated with post-flood resilience. Perceived social support was positively associated with resilience, and inversely correlated with PTSD symptoms. These data suggest that religiosity and perceived social support are valuable resources that foster post-disaster resilience among middle aged and older adults. Implications of these data for current views on age-related strengths and vulnerabilities after severe weather events are discussed.

